# 

**Published:** 1911-09

**Authors:** 


					CONTENTS. page
Med'cal Organisation and the Growth of the Medical Sciences
in the Seventeenth Century, Illustrated by the Lives of Local
Worthies.
By George Parker, M.A., M.D. Cantab.
The End-Results of Operations on the Stomach and Duodenum.
By A. Rendle Short, B.Sc., M.D. Lond., B.S., F.R.C.S.
Reviews of Books
256
Editorial Notes
278
Notes on Preparations for the Sick
28i
The Library of the Bristol Medico-Chirurgical Society ... ... 283
Obituary Notices ...
284
Local Medical Notes
287

				

